# Supplementary material for: Sacral agenesis: a pilot whole exome sequencing and copy number study
Source: BMC Med Genet. 2016 Dec 22;17:98. doi: 10.1186/s12881-016-0359-2 (PMC5178083; doi:10.1186/s12881-016-0359-2)
Supplement: Additional file 1: — Supplementary Methods and Material. (DOCX 51 kb) [file 12881_2016_359_MOESM1_ESM.docx]

# Sacral agenesis: A pilot whole exome sequencing and copy number study

Authors: Robert M. PORSCH, Elisa MERELLO, Patrizia DE MARCO, Guo CHENG, Laura RODRIGUEZ, Manting SO Pak C. SHAM, Paul K. TAM, Valeria CAPRA, Stacey S. CHERNY, Maria-Mercè GARCIA-BARCELO, Desmond D. CAMPBELL

Contents

[Supplementary Methods 1](#_Toc443492891)

[Quality control (QC) and variant calling 1](#_Toc443492892)

[CNV Analysis 2](#_Toc443492893)

[Supplementary Material 3](#_Toc443492894)

[References 4](#_Toc443492895)

# Supplementary Methods

## Quality control (QC) and variant calling

Initial quality assessment of raw reads was done using FastQC [1]. This included GC content, duplication levels and primer sequences. All sequenced individuals passed this initial quality control.

After aligment ,using BWA-MEM (version 0.7) [2], verifyBAMID [3] was used to exclude any potential contaminated samples. None of the analysed samples showed sign of contamination.

Calling of variants and indels was done using the GATK UnifiedGenotyper (Version) [4]. During the process of variant calling the number of false positives is generally high. Thus further quality control measures were taken in order to ensure valid calls. A hard filtering strategy to exclude false discovered variants was applied. We used the following filtering criteria suggested by GATK:

- QualByDepth (QD) 2.0
- FisherStrand (FS) 60.0
- RMSMappingQuality (MQ) 40.0
- MappingQualityRankSumTest (MQRankSum) 12.5
- ReadPosRankSumTest (ReadPosRankSum) 8.0

The alternative of a soft or self defining filtering strategy [5] was not optimal due to the low sample size.

Following we used KGGSeq [6] to hard filter variants of each individual by removing those variants with a coverage lower than 8 and a genotyping quality less than 20. KGGSeq also annotated variants by population frequency as provided by ESP6500 [7] as well as the 1000 Genome Project [8]. Relationship between participants was investigated using PLINK [9].

## CNV Analysis

Copy number variations (CNV) in all complete Italian families (CR541, CR17 and CR41) were detected using Illumina’s HumanCoreExome-24 beadchip. Quality control of the assayed genotypes was performed using GenomeStudio (Illumina Inc.) using the default settings. PennCNV[10] was used for trio-based CNV calling and *de novo* CNV detection. CNVs which do not overlap with CNVs seen in the 1000 Genome Project [8] were deemed rare. This is justified given that the chances of a rare variant being seen in the 1000 Genome Project data are high. For instance one would expect a variant with a minor allele frequency of 1e-4, 1e-3 or 2e-3 to occur in 1000 genomes respectively 18%, 86% and 98% of the time. In addition we also manually assessed the inheritance status of individual CNVs in order to confirm the results generated by PennCNV. *De novo* and homozygous CNVs are in Table 3 of the main manuscript. Rare CNVs are presented in Table 1.

# Supplementary Material I

## CNV

| Table 1. Rare CNVs | | | | | | |
| --- | --- | --- | --- | --- | --- | --- |
|  | **Chromosome** | **Start Position** | **End Position** | **Length** | **Type** | **Overlapping Genes** |
| CR5C |  |  |  |  |  |  |
|  | 11q14.1 | 81509575 | 81516733 | 7159 | deletion |  |
|  | 15q14 | 39196605 | 39331794 | 135190 | duplication | C15orf54, |
|  | 15q15.2-3 | 43252763 | 43322112 | 69350 | duplication | TGM7,ATP5HP1,TGM5 |
|  | 17q24.2 | 68485688 | 68506682 | 20995 | duplication |  |
|  | 2q36.3 | 228243905 | 228254272 | 10368 | duplication |  |
|  | 3q13.11 | 106351642 | 106355415 | 3774 | deletion |  |
|  | 9p24.1 | 7658953 | 7684244 | 25292 | deletion |  |
|  | 9q22.31 | 93557697 | 93573541 | 15845 | duplication | FAM120A |
|  | 9q22.31 | 93603137 | 93662762 | 59626 | duplication | PHF2 |
| CR17C |  |  |  |  |  |  |
|  | 10q21.3 | 68214970 | 68578970 | 364001 | deletion | DNA2,TET1,ATOH7,TMEM14DP,KRT19P4,SLC25A16,HNRNPH3,PBLD,RUFY2,RNA5SP319 |
|  | 14q11.2 | 20213937 | 20404091 | 190155 | duplication | OR11H7, OR11H4,PARP2,OR11H6,CCNB1IP1,SNORD126,RPPH1,TTC5,TEP1 |
|  | 20p12.3 | 5532853 | 5571964 | 39112 | duplication | GPCPD1,RPS18P1, EIF4EP1 |
| CR41C |  |  |  |  |  |  |
|  | 10q11.22 | 47543322 | 47703869 | 160548 | duplication | BMS1P2,FAM35DP,RN7SL453P,DUSP8P4,GLUD1P8,CTSLP2 |
|  | 11q14.1 | 81509575 | 81516733 | 7159 | deletion |  |
|  | 15q25.2 | 82028704 | 82096524 | 67821 | duplication | LINC01583,MEX3B, |
|  | 19p12 | 23364565 | 23408129 | 43565 | deletion | CDC42EP3P1,BNIP3P8,LINC01224,ZNF91 |
|  | 19p13.2 | 6961182 | 7014437 | 53256 | deletion | ADGRE4P |
|  | 5q22.2 | 113160490 | 1.13E+08 | 9956 | deletion | MCC |
|  | 5p13.3 | 32123891 | 32151119 | 27229 | duplication | GOLPH3 |

# References

1. Andrews S: **FastQC: A quality control tool for high throughput sequence data**. *babraham Bioinforma* 2010:1.

2. Li H: **Aligning sequence reads, clone sequences and assembly contigs with BWA-MEM**. 2013, **00**:3.

3. Jun G, Flickinger M, Hetrick KN, Romm JM, Doheny KF, Abecasis GR, Boehnke M, Kang HM: **Detecting and estimating contamination of human DNA samples in sequencing and array-based genotype data.** *Am J Hum Genet* 2012, **91**:839–48.

4. DePristo MA, Banks E, Poplin R, Garimella K V, Maguire JR, Hartl C, Philippakis AA, del Angel G, Rivas MA, Hanna M, McKenna A, Fennell TJ, Kernytsky AM, Sivachenko AY, Cibulskis K, Gabriel SB, Altshuler D, Daly MJ: **A framework for variation discovery and genotyping using next-generation DNA sequencing data.** *Nat Genet* 2011, **43**:491–8.

5. McKenna A, Hanna M, Banks E, Sivachenko A, Cibulskis K, Kernytsky A, Garimella K, Altshuler D, Gabriel S, Daly M, DePristo MA: **The Genome Analysis Toolkit: a MapReduce framework for analyzing next-generation DNA sequencing data.** *Genome Res* 2010, **20**:1297–303.

6. Li M-X, Gui H-S, Kwan JSH, Bao S-Y, Sham PC: **A comprehensive framework for prioritizing variants in exome sequencing studies of Mendelian diseases.** *Nucleic Acids Res* 2012, **40**:e53.

7. **Exome Variant Server** [http://evs.gs.washington.edu/EVS/]

8. Auton A, Abecasis GR, Altshuler DM, Durbin RM, Abecasis GR, Bentley DR, Chakravarti A, Clark AG, Donnelly P, Eichler EE, Flicek P, Gabriel SB, Gibbs R a., Green ED, Hurles ME, Knoppers BM, Korbel JO, Lander ES, Lee C, Lehrach H, Mardis ER, Marth GT, McVean G a., Nickerson D a., Schmidt JP, Sherry ST, Wang J, Wilson RK, Gibbs R a., Boerwinkle E, et al.: **A global reference for human genetic variation**. *Nature* 2015, **526**:68–74.

9. Purcell S, Neale B, Todd-Brown K, Thomas L, Ferreira MAR, Bender D, Maller J, Sklar P, de Bakker PIW, Daly MJ, Sham PC: **PLINK: a tool set for whole-genome association and population-based linkage analyses.** *Am J Hum Genet* 2007, **81**:559–75.

10. Wang K, Li M, Hadley D, Liu R, Glessner J, Grant SF a, Hakonarson H, Bucan M: **PennCNV: An integrated hidden Markov model designed for high-resolution copy number variation detection in whole-genome SNP genotyping data**. *Genome Res* 2007, **17**:1665–1674.
